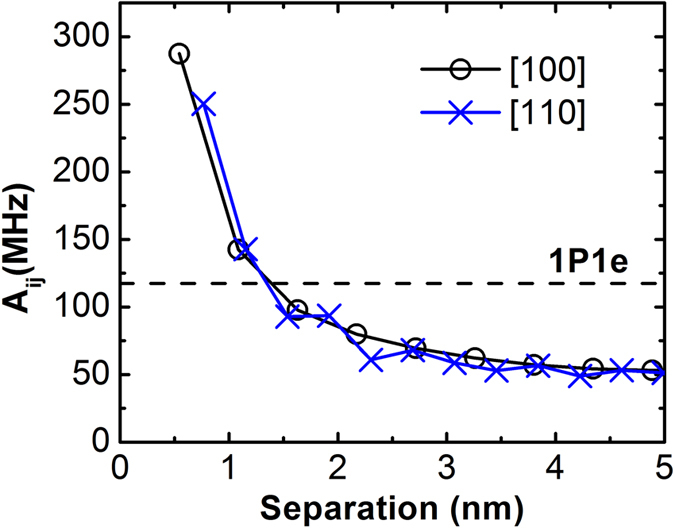# Corrigendum: Characterizing Si:P quantum dot qubits with spin resonance techniques

**DOI:** 10.1038/srep38120

**Published:** 2016-11-30

**Authors:** Yu Wang, Chin-Yi Chen, Gerhard Klimeck, Michelle Y. Simmons, Rajib Rahman

Scientific Reports 6: Article number: 3183010.1038/srep31830; published online: 08 23 2016; updated: 11 30 2016

This Article contains errors in Figure 4 where the hyperfine values for the donor separations along the [110] direction were calculated incorrectly. The correct Figure 4 appears below as Figure 1.

As a result, in the Results and Discussions section,

“From Fig. 4 we can see that *A*_*ij*_ of a P-donor pair in silicon with one bound electron can vary from ~366.0 MHz to ~48.9 MHz within a 5 nm separation range”.

should read:

“From Fig. 4 we can see that *A*_*ij*_ of a P-donor pair in silicon with one bound electron can vary from ~287.4 MHz to ~48.9 MHz within a 5 nm separation range”.

## Figures and Tables

**Figure 1:**