# Supplementary material for: Characterizing Si:P quantum dot qubits with spin resonance techniques
Source: Sci Rep. 2016 Aug 23;6:31830. doi: 10.1038/srep31830 (PMC4994117; doi:10.1038/srep31830)
Supplement: Supplementary Information [file srep31830-s1.pdf]

Supplemental information: Characterizing Si:P quantum dot qubits with spin resonance techniques

Yu Wang[1,*], Chin-Yi Chen[1], Gerhard Klimeck[1], Michelle Y. Simmons[2], & Rajib Rahman[1]

[1]*Network for Computational Nanotechnology, Purdue University, West Lafayette, IN 47907, USA*

[2]*Centre for Quantum Computation and Communication Technology, School of Physics, University of New South Wales, Sydney, NSW 2052, Australia*

Stark effect of ESR spectra of Si:P quantum dots under gate biases

Here we have investigated the effect of electric field (E-field) from a top gate, as in the typical Kane proposal[1–3], on the ESR spectra of Si:P quantum dots. The Si:P quantum dots are placed ~15 nm below the Si/SiO_2 interface (see Fig. 1(a) in the main text). As can be seen in Fig. S1(a), the change in the hyperfine couplings due to the electric fields is quite small for 1P (~9 MHz) and almost negligible for the 2P cases before the bound electron is ionized (where A_{ij} suddenly goes to 0). The corresponding shift in the ESR frequencies Δf_{ESR} (Fig. S1(b)) is 4MHz for the 1P case and ~1MHz for the 2P cases up to the ionization fields. Here we assume all the nuclear spins are in their lowest-energy configurations (all $\Uparrow$). Therefore the E-field induced shifts in the ESR frequencies are not comparable to the separation of the ESR frequencies, which are in the order of ~100MHz for the 1P and 2P1e cases, and ~10MHz for the 2P3e case as shown in the main text. This is because the electron wavefunction is strongly confined in a donor dot qubit and spatially small. The typical Bohr radius is 1-2 nm. As a result, the wavefunction is

not so sensitive to electric fields given by the top gates, so are the hyperfine interactions and the ESR spectra, because the fraction of electron wavefunction pulled away from the dot (toward the interface) is almost negligible. For STM-patterned devices, the electric fields can also be applied laterally with in-plane gates. We have shown in our previous work[4] that the stark shift of hyperfine interaction of a 2P dot is ∼10 MHz, and even smaller for a 3P dot with lateral electric fields up to 7 MV/m. Therefore, the Stark shift in the ESR frequencies does not play a significant role in the metrology proposed in this work. Moreover, the shift is predictable and can be accounted for when characterizing a realistic device under gate biases.

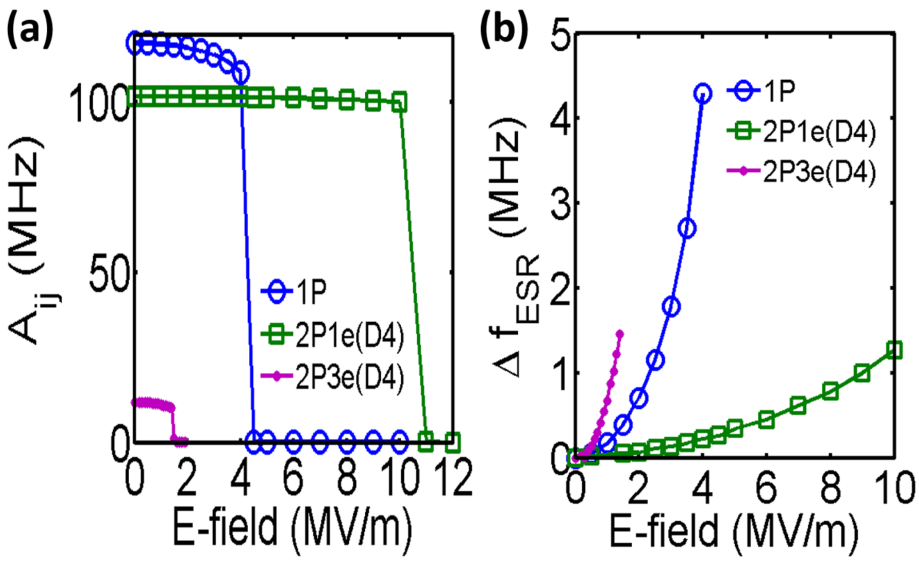

Figure S1: Electric field dependence of (a) Fermi contact hyperfine couplings A_{ij} (b) shift of ESR frequencies Δf_{ESR} with all nuclear spins $\Uparrow$.

1. Kane, B. E. A silicon-based nuclear spin quantum computer. Nature **393**, 133-137 (1998).

2. Rahman, R. *et al.* High precision quantum control of single donor spins in silicon. Phys. Rev. Lett. **99**, 036403 (2007).

3. Laucht, A. *et al.* Electrically controlling single-spin qubits in a continuous microwave field. Sci. Adv. 1, e1500022 (2015).

4. Büch, H., Mahapatra, S., Rahman, R., Morello, A. & Simmons, M. Y. Spin readout and addressability of phosphorus-donor clusters in silicon. Nat. Commun. **4**, 2017 (2013).
